# Supplementary material for: Seminoma and Embryonal Carcinoma Footprints Identified by Analysis of Integrated Genome-Wide Epigenetic and Expression Profiles of Germ Cell Cancer Cell Lines
Source: PLoS One. 2014 Jun 2;9(6):e98330. doi: 10.1371/journal.pone.0098330 (PMC4041891; doi:10.1371/journal.pone.0098330)
Supplement: File S1 — ZIP file containing DMRforPairs output for significant regions. Please start from the html files. (ZIP) [file pone.0098330.s008.zip › figures/7035.pdf]

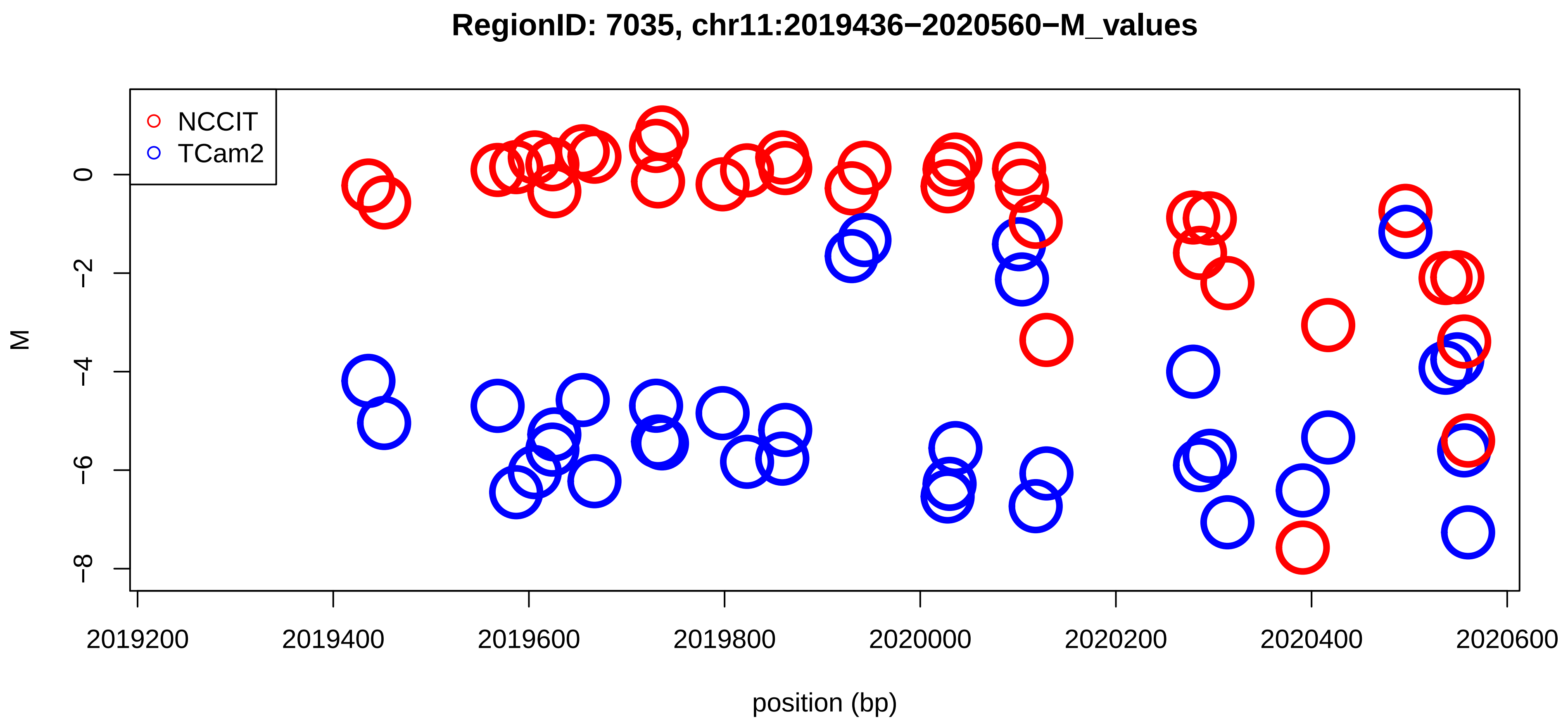

RegionID: 7035, chr11:2019436−2020560−M_values
NCCIT
TCam2
M
0
−2
−4
−6
−8
2019200
2019400
2019600
2019800
2020000
2020200
2020400
2020600
position (bp)

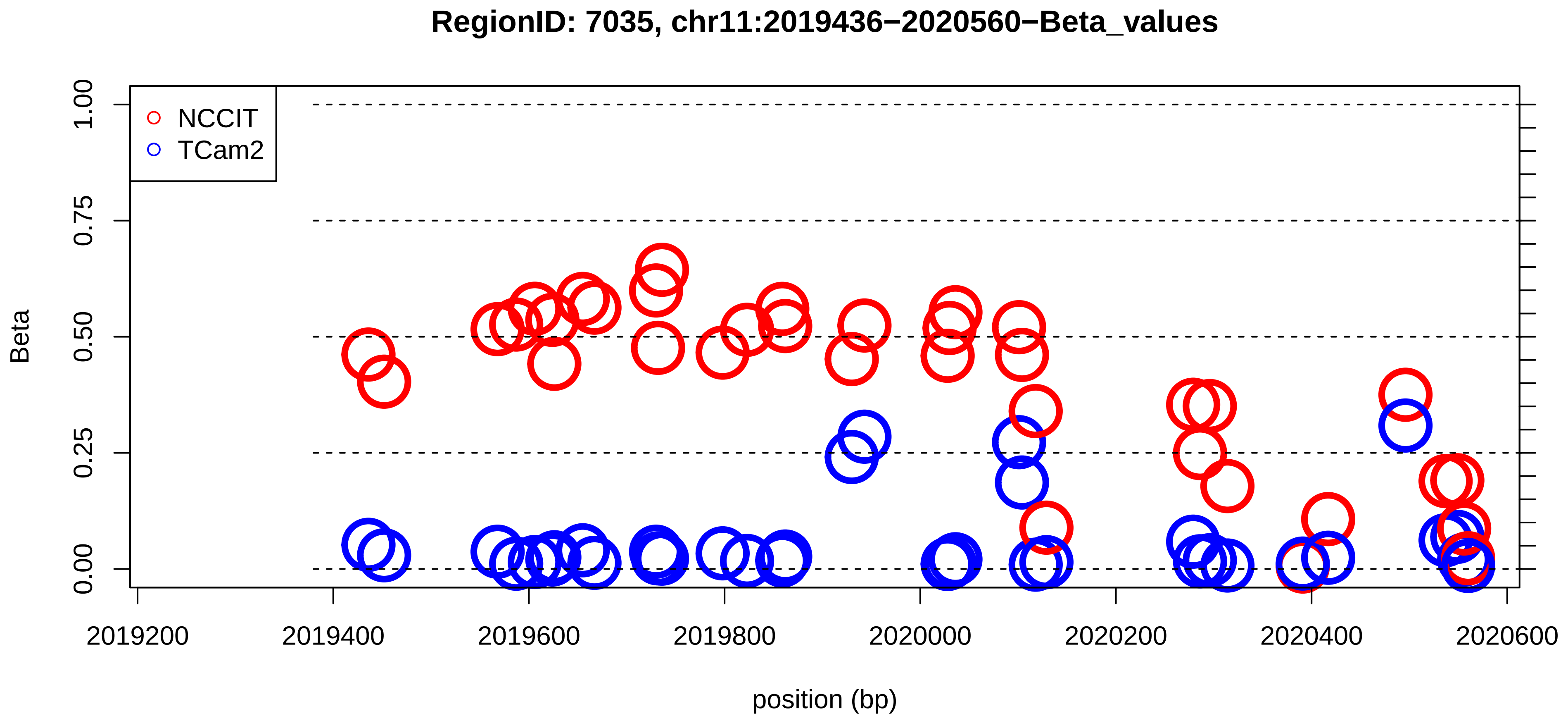

RegionID: 7035, chr11:2019436−2020560−Beta_values
NCCIT
TCam2
Beta
0.00
0.25
0.50
0.75
1.00
2019200
2019400
2019600
2019800
2020000
2020200
2020400
2020600
position (bp)

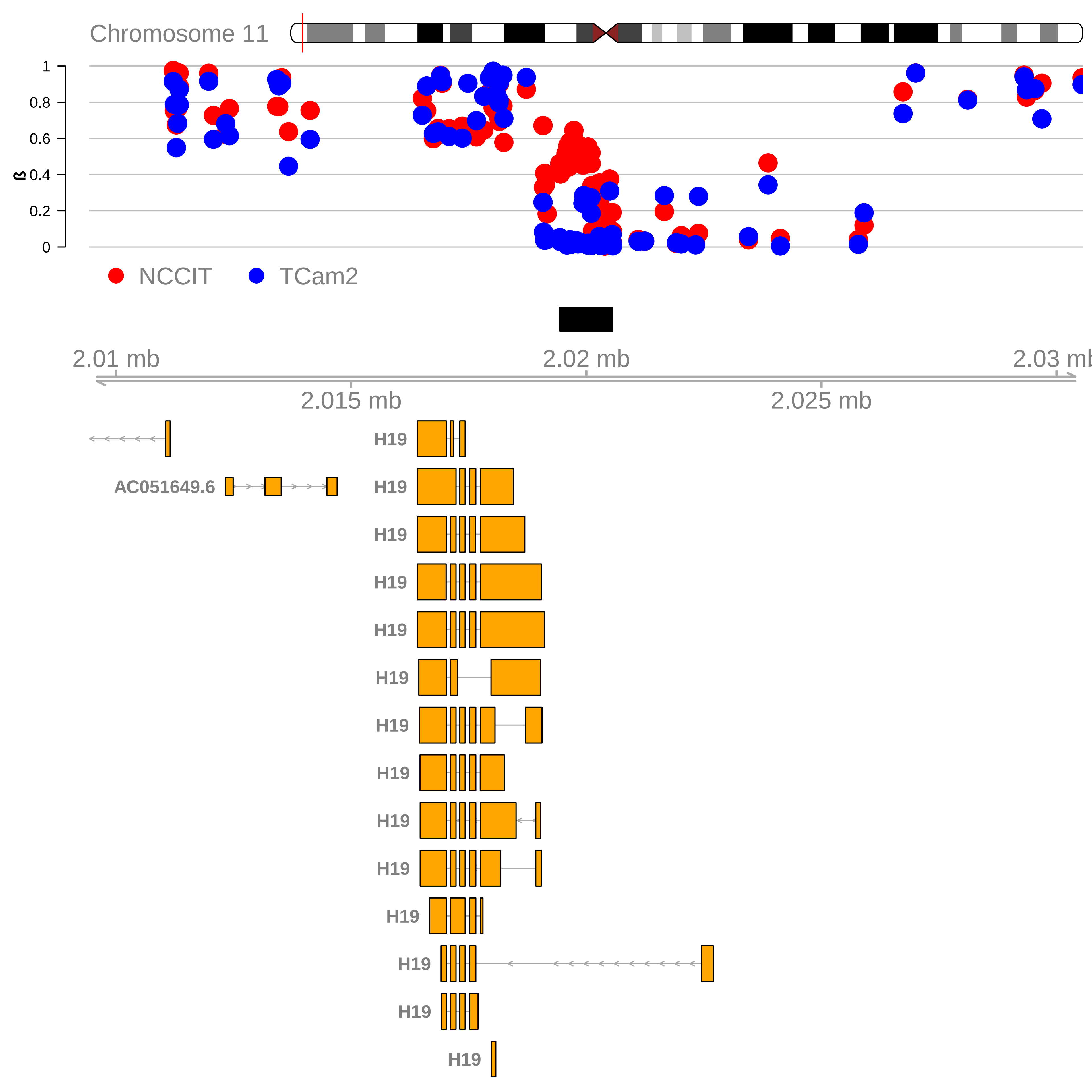
Chromosome 11
1
0.8
0.6
ß 0.4
0.2
0
NCCIT
TCam2
2.01 mb
2.02 mb
2.03 mb
2.015 mb
2.025 mb
AC051649.6
H19
H19
H19
H19
H19
H19
H19
H19
H19
H19
H19
H19
H19
H19
H19
